# A New Method to Evaluate Joint Hypermobility in Paediatric Patients with Neurodevelopmental Disorders: A Preliminary Study

**DOI:** 10.3390/children11091150

**Published:** 2024-09-23

**Authors:** Leonardo Zoccante, Marco Luigi Ciceri, Gianfranco Di Gennaro, Marco Zaffanello

**Affiliations:** 1 Childhood, Adolescence, Families and Family Health Center, Provincial Center for Autism Spectrum Disorders, 37122 Verona, Italy; leonardo.zoccante@aulss9.veneto.it (L.Z.); marco.ciceri@aulss9.veneto.it (M.L.C.); 2 Science of Health Department, School of Medicine, Magna Graecia University of Catanzaro, 88100 Catanzaro, Italy; gianfranco.digennaro@unicz.it; 3 Department of Surgery, Dentistry, Pediatrics and Gynecology, University of Verona, 37126 Verona, Italy

**Keywords:** attention-deficit/hyperactivity disorder, ADHD, autism, Beighton score, Brighton score, children, Tourette syndrome, passive ankle range of motion

## Abstract

**Background/Objectives**: Neurodevelopmental disorders (NDDs) include a wide range of conditions that develop during the formation of the central nervous system, such as autism spectrum disorder (ASD) and attention-deficit/hyperactivity disorder (ADHD). Tourette syndrome (TS) is another neurodevelopmental disorder characterised by motor and vocal tics, which often co-occurs with ASD and ADHD. This study explores the feasibility of assessing joint hypermobility in children with specific neurodevelopmental conditions by measuring both ankles’ passive range of motion (pROM). **Methods**: This study involved children diagnosed with ASD, ADHD, and TS, aged 5 to 15 years, who were compared with a control group of healthy children. The Beighton and Brighton scores and the pROM of the left and right ankles were measured. Data were analysed using SPSS version 22.0 for Windows (IBM SPSS Statistics, Chicago, IL, USA). A total of 102 subjects participated in this study (72.52% male, with a mean age of 10.7 ± 2.2 years). The sample included 24 children with ASD, 27 with ADHD, 26 with TS, and 25 healthy controls. **Results**: The pROM of the right and left ankles showed a significant positive correlation with the Beighton and Brighton scores in children with NDDs (ASD, ADHD, and TS combined). A trend towards higher Beighton scores (≥6) was observed in the ADHD and TS groups, with significance found in the TS group (*p* = 0.013). The pROM of the right ankle was significantly higher in the ADHD (*p* = 0.021) and TS (*p* = 0.013) groups compared to the controls. Although the left ankle followed a similar trend in the TS group, the difference was not statistically significant (*p* = 0.066). Controlling for age, the diagnosis of ASD, ADHD, and TS does not appear to impact any of the variables examined. **Conclusions**: There is a trend towards a higher prevalence of individuals with elevated Beighton scores in the ADHD and TS groups, suggesting greater general flexibility or hypermobility in these patients. However, the pROM of the right ankle is significantly higher in the ADHD and TS groups, with solid evidence in the TS group. These findings were not observed in children with ASD. However, it is necessary to consider the measurements obtained in relation to the patients’ age. Finally, given that the pROM of the ankles correlates with the Beighton and Brighton scores, it could be utilised for the initial screening, monitoring, and follow-up of JH in some children with NDDs. Further investigations are required.

## 1. Introduction

Neurodevelopmental disorders (NDDs) encompass conditions that emerge during the development of the central nervous system, affecting brain function and leading to difficulties in motor, cognitive, linguistic, and social skills. Autism spectrum disorder (ASD), attention-deficit/hyperactivity disorder (ADHD), and Tourette syndrome are all classified as NDDs according to the DSM-5 classification [1]. ASD, ADHD, and TS are frequently interconnected, with ADHD and ASD commonly co-occurring in individuals with TS, and ADHD and ASD often presenting together [2,3].

Recent research has highlighted a possible association between NDDs and connective tissue disorders [4], with alterations in connective tissue being observed in ASD [5,6]. Recent evidence also indicates the presence of structural and tissue changes [7]. These alterations may contribute to common symptoms and comorbidities, such as joint hypermobility (JH) and hypotonia, which are frequently observed in individuals with ASD [4,8]. Similar associations have been noted in ADHD, where individuals often exhibit generalised JH, also known as increased flexibility or joint laxity, with studies reporting that individuals with ADHD exhibit more generalised JH compared to the general population [9,10]. Given the overlap of these disorders, particularly in children with TS, understanding the underlying mechanisms is critical [1].

The Beighton and Brighton scores are tools used to assess JH and are particularly relevant in paediatric populations [11,12]. However, distinguishing between hypermobility, joint laxity, and muscle hypotonia, particularly in the ankle, remains clinically challenging due to the anatomical complexity of the region [13]. The ankle is crucial for postural control, and assessing its passive range of motion (pROM) can provide valuable insights into joint mobility without the confounding influence of muscle activation [14].

### Aims

This study aims to bridge the gap in understanding the relationship between certain NDDs (ASD, ADHD, and TS) and connective tissue alterations, with a focus on ankle joint mobility. The primary objective is to correlate the pROM of the ankles with Beighton and Brighton scores. The secondary objective is to assess the feasibility of measuring the pROM of the ankles in children with ASD, ADHD, and TS.

## 2. Materials and Methods

### 2.1. Subjects

This study involved 102 participants, all of whom had been previously diagnosed with ASD, ADHD, or TS and whose parents/guardians provided consent for their participation. The diagnosis of mild ASD, ADHD, or TS was made in clinical settings by specialists (LZ, MLC), following standard diagnostic criteria (DSM-5 or ICD-10). In particular, the assessments were carried out by clinicians specialising in neurodevelopmental disorders with extensive experience in diagnosing and managing conditions like ASD, ADHD, and TS. Assessors were not blinded to the diagnosis of the enrolled patients. Specifically, children aged 5 to 15 years with mild ASD, ADHD, or TS were approached for participation. In the medical history, a clinical history of trauma to the joints of the feet was ruled out.

All evaluations occurred in controlled clinical settings, specifically the Paediatric Clinic and the Child and Adolescent Neuropsychiatry Outpatient Clinics at the University Hospital of Verona. This approach ensured consistency and a standardised environment for all participants. Our study took place between December 2019 and November 2020, with all evaluations following the same protocol.

For the control group, data collection, including screenings for psychomotor and cognitive development, was conducted by independent clinicians to maintain impartiality in the assessment results. For the control group, children aged 5 to 15 years were consecutively enrolled from the COVID-19 vaccination clinic, ensuring they had no diagnosis of ASD, ADHD, TS, or other relevant medical conditions. Recruitment of controls was also carried out through the circulation of information flyers and by obtaining referrals from paediatricians. All participants in this group also underwent a preliminary assessment to confirm normal psychomotor and cognitive development and to rule out any psychiatric or neurological conditions. In the medical history, a clinical history of trauma to the joints of the feet was ruled out.

This study was conducted following the Declaration of Helsinki and was approved by the Institutional Review Board (or Ethics Committee) of the University Hospital of Verona (CESC 2243 (Paediatric Clinic, University Hospital of Verona) and CESC 2242 (Child and Adolescent Neuropsychiatry Outpatient Clinics, University Hospital of Verona)) on 10 December 2019. Informed consent was obtained from all participants and their legal guardians before the commencement of the study.

### 2.2. Beighton Scale

The Beighton scale is a nine-point assessment system used to evaluate JH, particularly generalised JH. The Beighton score involves a series of manoeuvres to assess joint flexibility in the elbows, knees, fingers, wrists, and lower back [11]. A Beighton score of ≥4 out of 9 is commonly used as a threshold to diagnose generalised JH [15].

Hypermobility has consistently been observed to be more common in younger children, with its prevalence decreasing as age increases. It declines rapidly during childhood (35.6% at age 10) and more gradually during adolescence (11.7% in children aged 13–19), often leading to overdiagnosis of generalised JH in younger age groups [11]. Finally, Smits-Engelsman et al. recommended a Beighton score threshold of 7/9 for Dutch children aged 6 to 12 years [16].

### 2.3. Brighton Scale

The Brighton scale is a standard screening tool for generalised JH and can assess whether ligamentous laxity is present in more than one body area [17]. The Brighton scale assesses JH and includes a combination of criteria beyond the Beighton score. It includes major and minor criteria for diagnosing Benign Joint Hypermobility Syndrome (BJHS) [12]. The Brighton scale also considers the metacarpophalangeal (MCP) joint extension angle of the 5th finger beyond 90 degrees, thumb abduction to the forearm, elbow hyperextension beyond 10 degrees, knee hyperextension beyond 10 degrees, and the ability to place the palms of the hands on the floor with straight legs. Each finding is awarded 1 point, and a score of ≥4 points is used to diagnose joint laxity [12].

### 2.4. Passive Ankle Range of Motion (pROM)

The measurement of the pROM of the ankle involves assessing the movement of the ankle joint in both dorsiflexion (upward movement) and plantarflexion (downward movement). The patient sits with their leg on the edge of the examination table or bed. The examination stabilises the ankle, ensuring the foot is at a right angle to the tibia and not resting on the floor.

The goniometer is placed on the lateral malleolus (the bony prominence outside the lower leg). The fixed arm is aligned parallel to the fibula (the outer bone of the lower leg). The movable arm points towards the fifth toe.

For dorsiflexion, the examiner raises the foot towards the tibia, and the goniometer measures the angle between the plantar surface (ground) and the posterior cortical surface of the tibia (bone of the lower leg). For plantarflexion, the examiner moves the foot away from the tibia, and the goniometer measures the angle in the opposite direction [18,19,20].

### 2.5. Statistical Analyses

The data were recorded in a Microsoft^®^ Excel^®^ 2408 version database for Windows 11 and statistically analysed using SPSS version 22.0 for Windows (SPSS Inc., Chicago, IL, USA). Statistical analyses were expressed as *n* (%) and mean (SD) years, minimum and maximum. Mean age and standard deviation (SD) are provided for each diagnostic group.

The Shapiro–Wilk test was used to assess the normality of continuous variables. A *p*-value <0.05 suggests that the data significantly deviate from a normal distribution. Continuous variables were compared using Fisher’s exact test, expressed as mean ± SD (95% CI) for independent samples; ANOVA if more than two groups were considered simultaneously; and the non-parametric chi-square test for comparing proportions.

Partial correlation analysis was used to examine the strength and direction of the relationship between two variables while accounting for one or more confounding factors. A *p*-value <0.05 was considered statistically significant. However, we recognise that the *p*-value in our study could be affected by factors like the small sample size, potential bias, and random error. Therefore, we will also consider potential statistical significance with a *p*-value between 0.05 and 0.1, explaining this value and other evidence supporting the relationship [21].

## 3. Results

Table 1 describes the sample consisting of 102 participants, divided into four groups: mild ASD (*n* = 24; 10.7 ± 2.2 years), ADHD (*n* = 27; 9.7 ± 2.2 years), TS (*n* = 26; 12.0 ± 1.8 years), and the controls *(n* = 25; 12.0 ± 2.2 years).

There is a statistically significant difference in the distribution of males between the groups (Pearson’s chi-square: 18.8; *p* < 0.001) and a statistically significant difference in mean ages between the groups (ANOVA, *p* < 0.001). Significant differences between the four groups of subjects were found only for the pROM of the right ankle (ANOVA, *p* = 0.019).

The Shapiro–Wilk test indicates that the Beighton score, Brighton score, and pROM for both the right and left ankles do not follow a Gaussian distribution for the entire group of patients examined. The ages of the participants are normally distributed (*p* < 0.001).

Age at the visit was correlated with the Beighton and Brighton scores and the pROM of the right and left ankles, controlling for sex. An increase in age was associated with a significant reduction in the Beighton score (r = −0.386; *p* < 0.001), the Brighton score (r = −0.290; *p* = 0.003), and the pROM of the right ankle (r = −0.488; *p* < 0.001) and the left ankle (r = −0.435; *p* < 0.001).

Table 2 examines the partial correlations between the pROM of the right and left ankles and the Beighton and Brighton scores, adjusted for age and sex. For example, considering subjects with ASD, ADHD, and TS together, the pROM of the right ankle showed a statistically significant correlation with the generalised JH scores assessed with the Beighton score (r = 0.327; *p* = 0.004) and Brighton score (r = 0.284; *p* = 0.013).

Additionally, the pROM of the left ankle showed a significant positive correlation with the generalised JH scores assessed with Beighton (r = 0.337; *p* = 0.003) and Brighton (r = 0.273; *p* = 0.018).

For the controls, the pROM of the right and left ankles did not significantly correlate with the JH assessed by the Beighton score. Furthermore, the pROM of the right ankle correlated with the Brighton score (r = 0.429; *p* = 0.041) but not the pROM of the left ankle after adjusting for age and sex.

**Figure 1:**
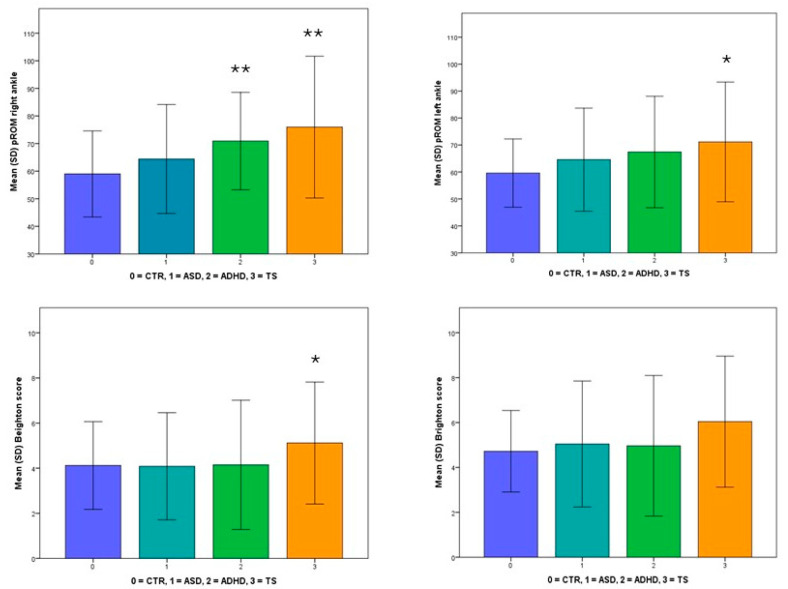
The mean values (SD) of the Brighton and Beighton scores and the pROM of the right and left ankles in children with ASD, ADHD, or TS and the healthy controls. Legend: ADHD, attention-deficit/hyperactivity disorder; ASD, autism spectrum disorder; CTR, control; TS, Tourette syndrome. **, statistically significant; * *p* > 0.05 < 0.1.

Table 3 and Figure 1 show the Brighton and Beighton scores’ mean (SD) values. There are no significant differences in the mean Beighton and Brighton scores between the ASD, ADHD, and TS groups compared to the controls (*p* = NS). The proportion of patients with a Beighton score of ≥4 is similar across the groups, with no significant differences. However, there is a trend towards a higher prevalence of individuals with higher Beighton scores (≥6) in the ADHD and TS groups, with significance observed for the TS group (*p* = 0.013). Additionally, there is a trend towards higher Brighton scores (≥7) in the clinical groups, with significance noted for the TS group (*p* = 0.016).

Table 3 and Figure 1 also show the mean (SD) values of pROM for the right and left ankles. The mean pROM for the right ankle is higher in the clinical groups than the controls, with a significant difference in the ADHD group (*p* = 0.021) and TS group (*p* = 0.013). The pROM for the left ankle follows a similar trend, with marginal significance observed for the TS group (*p* = 0.066).

The multiple regression analysis (Table 4) indicates that the age at the visit (as a covariate) significantly affects most dependent variables, except for the Brighton score in ASD and TS and the pROM of the left ankle in TS. The diagnosis of ASD, ADHD, and TS does not appear to affect any of the variables examined. In particular, the findings suggest that in subjects with ADHD, age significantly influences the dependent variables. The results suggest that age in ASD significantly affects the dependent variables except for the Brighton score. The diagnosis of TS does not significantly influence the dependent variables. Therefore, age significantly affects the Beighton score and the pROM of the right ankle, with a marginally significant effect on the Brighton score but no significant impact on the pROM of the left ankle.

## 4. Discussion

The pROM of the right and left ankles shows significant correlations with the Beighton and Brighton scores in all of our patients with NDDs, including ASD, ADHD, and TS. In contrast, no correlation was observed in the control group.

The mean Beighton and Brighton scores do not show significant differences between the groups and the healthy controls. Nevertheless, there is a significantly higher prevalence of elevated Beighton score (≥6; *p* = 0.013) and Brighton score (≥7; *p* = 0.016) in the TS group than in the healthy controls. Additionally, there is a trend towards a higher prevalence of Beighton and Brighton scores in the ADHD group. However, the study reveals a significantly higher pROM of the right ankle in patients with ADHD (*p* = 0.021) and TS (*p* = 0.013) compared to the healthy controls. However, no significant differences were found in children with ASD.

Adjusting for age, the diagnosis of ASD, ADHD, and TS does not appear to impact any of the variables examined (Beighton and Brighton scores and pROM). However, the significant effect of age suggests that it is an essential factor that must be considered. However, the significant results from the non-parametric test on the pROM of the right ankle in the ADHD and TS groups highlight the relevance of age for certain variables but not for all.

Previous research has reported that adults (18–61 years) with ASD (4.5 times higher), ADHD (4.3 times higher), and TS (7.0 times higher) have generalised JH [10,22].

Previous studies suggest that a Beighton score of ≥4 is not sufficiently sensitive to detect differences between study groups, in contrast to a threshold score of ≥6. Consequently, Smits-Engelsman et al. proposed a higher threshold for the Beighton score in the paediatric age group [16]. Indeed, there was a trend towards a higher prevalence of elevated Beighton scores (≥6) in the ADHD and TS groups, with statistical significance observed for TS (*p* = 0.013) but not for ADHD children (*p* = 0.093). Accordingly, the threshold for identifying hypermobility in children has been reported to be at least ≥6 on the Beighton score [10,23].

Using the Brighton score (≥7), statistical significance was observed for TS (*p* = 0.016), and there was a trend towards a higher prevalence of scores ≥7 in children with ADHD (*p* = 0.066). However, these results were not confirmed in our children with ASD.

Recent research findings indicate that hyperlaxity, measured using the Beighton scale, frequently coexists with ADHD and ASD [24]. Excessive hypermobility has subsequently been demonstrated in children with ASD, with a mean age of 4 years and 6 months [22]. Furthermore, a large-scale cross-sectional study revealed a significant relationship between ASD and generalised JH in adults. Logistic regression models, adjusted for covariates (age, sex, ethnicity), showed a significant association between ASD and generalised JH (OR 3.1, *p* < 0.001) [24]. The research shows that ADHD is often present alongside ASD, and this association could influence the relationship between ASD and generalised JH. One study suggests that ADHD might be a critical factor in this relationship [9].

In our sample of children, a solid and significant correlation was found between the Beighton and Brighton scores as measures of generalised JH [11,12] and the pROM of the right and left ankles after adjusting for age and sex.

The pROM of the right ankle shows significant associations with ADHD and TS. In TS, this condition can frequently be due to its comorbidity with ASD and ADHD [10]. Specifically, the pROM of the right ankle had higher mean values in patients with ADHD (*p* = 0.021) and TS (*p* = 0.013) compared to the healthy controls. Additionally, we found borderline significance for the pROM of the left ankle in subjects with TS (*p* = 0.066) compared to the healthy controls. However, data concerning hypermobility in children with TS are lacking [24]. Although information on hyperlaxity in TS is scarce, one study found a high prevalence (38%) of hypermobility in adults [24].

Hypermobility is a frequent sign of hereditary disorders of connective tissue [25]. This suggests a possible role for connective tissue disorders in clinical conditions such as ASD, ADHD, and TS [24], highlighting the need for further research into the interaction between joint flexibility and these neuropsychiatric disorders.

A total of 63% of patients with ASD aged between 2 and 4 years and 73% of children with ASD aged 5 years and older showed significant hypermobility scores [26]. Our study did not demonstrate generalised JH and JH of the ankle in children with ASD. The discrepancy between our findings on children with ASD and those reported in the literature may be due to the mild severity of ASD, the small sample size, and the absence of ADHD clinical features. However, toe walking is a frequent observation in ASD, and the pathological gait caused by bilateral myotendinous retraction of the calf muscles can transform an “equinus attitude” into a gait pattern typical of clubfoot. This posture may impact the objective assessment of JH [27]. Accordingly, toe walking may affect the objective evaluation of JH using the pROM, leading to a potential underestimation of joint mobility due to compensatory mechanisms or muscle rigidity associated with this gait pattern [28].

The limitations of this study include a significant difference in the average age between the study groups and the healthy controls, with the controls being older than the patient groups (ADHD and TS). Younger children tend to show higher levels of joint laxity than older children and adolescents [29]. The greater JH of the right ankle in the ADHD and TS groups compared to the controls can partly be explained by the younger age of the children with ADHD and TS, along with a possible association between neuropsychiatric disorders and a higher prevalence of JH. However, the inclusion criteria comprised the same age range, from 5 to 15 years, for all groups, minimising the age effect on the results obtained. Finally, the significant results of the non-parametric tests and Fisher’s test highlight the relevance of age for certain variables but not for all, suggesting that the age differences within the patient groups do not introduce significant random error or lead to erroneous findings.

In our study, we found a higher prevalence of males in the ASD, ADHD, and TS categories compared to the controls. Regarding gender, patients with ASD and ADHD showed a higher prevalence of males (75% and 88.9%, respectively) compared to patients with TS (84.6%) and the controls (40%; *p* < 0.001). A review reported that JH was more prevalent in females (32.5%) than males (18.1%) in children and adolescents. This study comprises a wide age range (3–19 years), and when combined with studies with significant heterogeneity, it may limit generalisation [29]. Therefore, as it has been reported that females might exhibit more significant ligamentous laxity than males, the imbalance favouring females in the control group could result in an underestimation (rather than an overestimation) of the results. However, in the statistical analysis, we adjusted for gender and age as covariates.

Another limitation of our study was the lack of measurement of the participants’ weight and height. The relationship between body weight or BMI and joint laxity is not well-established to date [23,30] or may only be associated with specific populations [31]. Future studies should include these measurements for a more comprehensive analysis. Additionally, a comparative analysis with other accepted methodologies is needed to further validate our proposed approach’s accuracy.

The strength of this study lies in the assessment of JH in the ankle using a novel method (pROM), which is compared with Beighton and Brighton scores across three groups of neurodivergent paediatric patients and a control population [31]. Immobilising the other joints of the lower limb while leaving only the ankle free could allow for the isolation of the tendinous component during measurement [32,33], unlike what occurs with other items on the Beighton scale. The choice to measure the pROM of the ankle, a joint crucial for gait and postural stability and not included in the Beighton scale, may justify its use.

## 5. Conclusions

The results indicate that the pROM of the right and left ankles shows a significant positive correlation with generalised JH measured using the Beighton and Brighton scales. Additionally, there is a trend towards a higher prevalence of individuals with elevated Beighton scores in the ADHD and TS groups, with significance observed for the TS group, suggesting greater general flexibility or hypermobility in these patients compared to the healthy controls. However, the pROM of the right ankle is significantly higher in the ADHD and TS groups, indicating increased joint mobility. These findings were not observed in children with ASD. However, it is necessary to consider the measurements obtained in relation to the patients’ age, as the age at the time of the visit might significantly affect both the scores and the pROM. Finally, given that the pROM of the ankles correlates with the Beighton and Brighton scores, it could be utilised for the initial screening, monitoring, and follow-up of JH in selected children with NDDs. Further investigations are required.

## Figures and Tables

**Table 1:** A description of the subjects included in this study, categorised by condition: mild autism spectrum disorder (ASD), attention-deficit/hyperactivity disorder (ADHD), Tourette syndrome (TS), and a control group. For each group, the total number of subjects (% male), mean age (SD) and age range, Beighton and Brighton scores, and passive range of motion (pROM) for the right and left ankles are reported. Finally, the results of the Shapiro–Wilk test are presented to assess the normal distribution of the measurements.

Variable	*n*. (% Total)	Males, %	Pearson’s Chi-Square (*p*)	Age, Mean (SD) Years	Minimum–Maximum	ANOVA (*p*)
*n*.	102	74 (72.5)		10.7 (2.2)	5.7–15.3	
ASD, *n*. (% total)	24 (23.5)	18 (75.0)		10.9 (2.0)	7.2–14.6	
ADHD, *n*. (% total)	27 (26.5)	24 (88.9)		9.7 (2.2)	5.7–14.4	
TS, *n*. (% Total)	26 (25.5)	22 (84.6)		10.2 (1.9)	7.4–14.5	
Controls, *n*. (% total)	25 (24.5)	73.2 (40)	18.89 (*p* < 0.001)	12.0 (1.8)	8.3–15.3	<0.001
	Mean	SD	Minimum–maximum	Shapiro–Wilk Test (Sign. asymptotic, two-tailed) *	95% C.I.	ANOVA (ASD, ADHD, TS, Controls)
Age, years	10.7	2.2	5.7–15.3	0.191	10.29–11.13	<0.001
Beighton score	4.47	2.5	0–9	<0.001	3.88–4.87	0.386
Brighton score	5.24	2.7	0–11	0.023	4.66–5.73	0.324
pROM right ankle	68.4	20.4	11–160	0.001	63.67–71.84	0.019
pROM left ankle	65.2	19.3	30–140	<0.001	62.0–69.57	0.181

Legend: ADHD, attention-deficit/hyperactivity disorder; ASD, autism spectrum disorder; pROM, passive range of motion; SD, standard deviation; TS, Tourette syndrome. (*) If *p* < 0.05, the distribution is not normal (does not follow a Gaussian distribution).

**Table 2:** Partial correlations between the generalised joint hypermobility scores, as measured by the Beighton and Brighton scores, and the pROM of the right and left ankles.

Partial Correlation Analysis (All Subjects)	Beighton Score r (*p*-Value)	Brighton Score r (*p*-Value)
Adjusted for age (years) and sex		
pROM right ankle	0.319 (0.001)	0.309 (0.002)
pROM left ankle	0.334 (0.001)	0.282 (0.004)
Partial correlation analysis (controls)		
Adjusted for age (years) and sex		
pROM right ankle	0.379 (0.074)	0.429 (0.041)
pROM left ankle	0.270 (0.213)	0.274 (0.206)
Partial correlation analysis (ASD, ADHD, TS)		
Adjusted for age (years) and sex		
pROM right ankle	0.327 (0.004)	0.284 (0.013)
pROM left ankle	0.337 (0.003)	0.273 (0.018)
Partial correlation analysis (ADHD, TS)		
Adjusted for age (years) and sex		
pROM right ankle	0.381 (0.006)	0.368 (0.008)
pROM left ankle	0.404 (0.003)	0.393 (0.004)

Legend: ADHD, attention-deficit/hyperactivity disorder; ASD, autism spectrum disorder; pROM, passive range of motion; TS, Tourette syndrome.

**Table 3:** Comparison of clinical and functional parameters between controls and groups with ASD, ADHD, and TS (ANOVA or Fisher’s exact test).

	Controls	ASD	Test U Mann–Whitney (ASD vs. Controls; *p*-Value)	Fisher’s (*p*-Value)	ADHD	Test U Mann–Whitney (ADHD vs. Controls; *p*-Value)	Fisher’s (*p*-Value)	TS	Test U Mann–Whitney (TS vs. Controls; *p*-Value)	Fisher’s (*p*-Value)	Test Kruskal–Wallis (ASD plus ADHS plus TS vs. Controls *p*-Value)	Chi-Square Pearson (ASD plus ADHS plus TS vs. Controls; *p*-Value)
*n*	25	24			27			26			102	
Age, mean (SD)	12.0 (1.8)	11.0 (2.0)	0.063		9.7 (2.2)	<0.001		10.2 (1.9)	0.003		0.001	
Beighton score, mean ± SD (95% CI) *	4.1 ± 1.9 (3.3–4.9)	4.1 ± 2.4 (3.1–5.1)	0.984		4.15 ± 2.9 (3.0–5.3)	0.948		5.1 ± 2.7 (4.0–6.2)	0.090		0.353	
Beighton score (≥4), *n* (%)	17 (68.0)	15 (62.5)		0.458	16 (59.3)		0.358	18 (69.2)		0.582		0.754 (0.860)
Beighton score (≥5), *n* (%)	11 (44.0)	9 (37.5)		0.432	11 (40.7)		0.517	17 (65.4)		0.105		4.912 (0.178)
Beighton score (≥6), *n* (%)	5 (20.0)	8 (33.3)		0.232	11 (40.7)		0.093	14 (53.8)		0.013		6.544 (0.088)
Beighton score (≥7), *n* (%)	2 (8.7)	4 (16.7)		0.314	7 (30.4)		0.089	10 (43.5)		0.011		7.451 (0.059)
Brighton score, mean ± SD (95% CI) *	4.7 ± 1.8 (4.0–5.5)	5.0 ± 2.8 (3.9–6.2)	0.578		5.0 ± 3.1 (3.7–6.2)	0.732		6.0 ± 2.9 (4.9–7.2)	0.062		0.330	
Brighton score (≥4), *n* (%)	18 (72.0)	18 (75)		0.534	17 (63.0)		0.346	21 (80.8)		0.342		2.204 (0.531)
Brighton score (≥5), *n* (%)	15 (60.0)	15 (62.5)		0.545	16 (59.3)		0.590	17 (65.4)		0.457		0.254 (0.968)
Brighton score (≥6), *n* (%)	9 (36.0)	10 (41.7)		0.455	13 (48.1)		0.273	16 (61.5)		0.061		3.708 (0.295)
Brighton score (≥7), *n* (%)	3 (10.3)	7 (29.2)		0.128	9 (31.0)		0.066	11 (37.9)		0.016		5.934 (0.115)
pROM right ankle, mean ± SD (95% CI)	59.0 ± 15.6 (52.6–5.4)	64.4 ± 19.7 (56.1–72.8)	0.285		70.9 ± 17.7 (63.9–77.9)	0.021		76.0 ± 25.7 (4.9–7.2)	0.013		0.044	
pROM left ankle, mean ± SD (95% CI)	59.6 ± 12.7 (54.4–64.8)	64.6 ± 19.1 (56.5–72.7)	0.493		67.2 ± 21.0 (58.9–75.5)	0.268		71.2 ± 22.2 (62.2–80.1)	0.066		0.319	

Legend: ADHD, attention-deficit/hyperactivity disorder; ASD, autism spectrum disorder; CI, Confidence interval; pROM: passive range of motion; SD, standard deviation; TS, Tourette syndrome. Yellow colour, statistically significant; light blue colour, *p* > 0.05 < 0.1. * Distribution of data is normal (Single-sample Kolmogorov–Smirnov test).

**Table 4:** The table presents the results of the multivariate analysis with the age at the visit as a covariate for the dependent variables Beighton, Brighton, and pROM of the right and left ankles. Comparisons are made between the ASD, ADHD, and TS groups and the control group.

					Covariate (Age)			
	Dependent Variable	Quadratic Mean	F	*p*-Value	Quadratic Mean	F	*p*-Value	Square R^2^
ASD vs. CTR	Beighton score	2.758	0.671	0.417	31.441	7.651	0.008	0.143
	Brighton score	0.257	0.046	0.831	4.401	0.792	0.378	0.002
	pROM right ankle	24.724	0.095	0.760	2302.774	8.471	0.006	0.176
	pROM left ankle	27.540	0.121	0.730	1766.210	7.756	0.008	0.165
ADHD vs. CTR	Beighton score	11.908	2.291	0.137	49.341	9.492	0.003	0.162
	Brighton score	5.651	0.938	0.338	38.693	6.420	0.015	0.118
	pROM right ankle	70.034	0.321	0.574	3257.347	14.924	0.000	0323
	pROM left ankle	13.749	0.057	0.813	3091.407	12.758	0.001	0.246
TS vs. CTR	Beighton score	0.290	0.058	0.810	34.589	7.018	0.011	0.166
	Brighton score	4.971	0.876	0.354	19.614	3.456	0.069	0.133
	pROM right ankle	1029.327	2.481	0.122	2437.985	5.877	0.019	0.235
	pROM left ankle	781.117	2.379	0.130	400.151	1.219	0.275	0.0118

Legend: ADHD, attention-deficit/hyperactivity disorder; ASD, autism spectrum disorder; CTR, control; pROM: passive range of motion; TS, Tourette syndrome.

## Data Availability

Data are unavailable due to privacy and ethical restrictions.

## References

[1] Cravedi E., Deniau E., Giannitelli M., Xavier J., Hartmann A., Cohen D. (2017). Tourette syndrome and other neurodevelopmental disorders: A comprehensive review. Child Adolesc. Psychiatry Ment. Health.

[2] Huisman-van Dijk H.M., Schoot R., Rijkeboer M.M., Mathews C.A., Cath D.C. (2016). The relationship between tics, OC, ADHD and autism symptoms: A cross-disorder symptom analysis in Gilles de la Tourette syndrome patients and family-members. Psychiatry Res..

[3] Cainelli E., Bisiacchi P. (2022). Neurodevelopmental Disorders: Past, Present, and Future. Children.

[4] Zoccante L., Ciceri M.L., Gozzi L.A., Gennaro G.D., Zerman N. (2021). The “Connectivome Theory”: A New Model to Understand Autism Spectrum Disorders. Front. Psychiatry.

[5] Hughes M.M., Shaw K.A., DiRienzo M., Durkin M.S., Esler A., Hall-Lande J., Wiggins L., Zahorodny W., Singer A., Maenner M.J. (2023). The Prevalence and Characteristics of Children with Profound Autism, 15 Sites, United States, 2000–2016. Public Health Rep..

[6] Baeza-Velasco C., Grahame R., Bravo J.F. (2017). A connective tissue disorder may underlie ESSENCE problems in childhood. Res. Dev. Disabil..

[7] Minshew N.J., Williams D.L. (2007). The new neurobiology of autism: Cortex, connectivity, and neuronal organisation. Arch. Neurol..

[8] Veronese S., Zoccante L., Smania N., Sbarbati A. (2023). Stretch marks: A visible expression of connective’s involvement in autism spectrum disorders. Front. Psychiatry.

[9] Glans M.R., Thelin N., Humble M.B., Elwin M., Bejerot S. (2021). The Relationship Between Generalised Joint Hypermobility and Autism Spectrum Disorder in Adults: A Large, Cross-Sectional, Case Control Comparison. Front. Psychiatry.

[10] Csecs J.L.L., Iodice V., Rae C.L., Brooke A., Simmons R., Quadt L., Savage G.K., Dowell N.G., Prowse F., Themelis K. (2021). Joint Hypermobility Links Neurodivergence to Dysautonomia and Pain. Front. Psychiatry.

[11] Malek S., Reinhold E.J., Pearce G.S. (2021). The Beighton Score as a measure of generalised joint hypermobility. Rheumatol. Int..

[12] Grahame R., Bird H.A., Child A. (2000). The revised (Brighton 1998) criteria for the diagnosis of benign joint hypermobility syndrome (BJHS). J. Rheumatol..

[13] Wilson A., Lichtwark G. (2011). The anatomical arrangement of muscle and tendon enhances limb versatility and locomotor performance. Philos. Trans. R. Soc. Lond. B Biol. Sci..

[14] Cho K.H., Jeon Y., Lee H. (2016). Range of Motion of the Ankle According to Pushing Force, Gender and Knee Position. Ann. Rehabil. Med..

[15] Beighton P., Horan F. (1969). Orthopaedic aspects of the Ehlers-Danlos syndrome. J. Bone Jt. Surg. Br..

[16] Smits-Engelsman B., Klerks M., Kirby A. (2011). Beighton score: A valid measure for generalised hypermobility in children. J. Pediatr..

[17] Remvig L., Jensen D.V., Ward R.C. (2007). Are diagnostic criteria for general joint hypermobility and benign joint hypermobility syndrome based on reproducible and valid tests? A review of the literature. J. Rheumatol..

[18] Tavares P., Landsman V., Wiltshire L. (2017). Intra-examiner reliability of measurements of ankle range of motion using a modified inclinometer: A pilot study. J. Can. Chiropr. Assoc..

[19] Dimakopoulos R., Syrogiannopoulos G., Youroukos S., Dailiana Z., Spinou A. (2019). Passive range of motion changes in young children with spastic diplegia. A study during the initial stages of independent walking. J. Pediatr. Rehabil. Med..

[20] Youn P.S., Cho K.H., Park S.J. (2020). Changes in Ankle Range of Motion, Gait Function and Standing Balance in Children with Bilateral Spastic Cerebral Palsy after Ankle Mobilization by Manual Therapy. Children.

[21] Thiese M.S., Ronna B., Ott U. (2016). P value interpretations and considerations. J. Thorac. Dis..

[22] Shetreat-Klein M., Shinnar S., Rapin I. (2014). Abnormalities of joint mobility and gait in children with autism spectrum disorders. Brain Dev..

[23] Williams C.M., Welch J.J., Scheper M., Tofts L., Pacey V. (2024). Variability of joint hypermobility in children: A meta-analytic approach to set cut-off scores. Eur. J. Pediatr..

[24] Sharp H.E.C., Critchley H.D., Eccles J.A. (2021). Connecting brain and body: Transdiagnostic relevance of connective tissue variants to neuropsychiatric symptom expression. World J. Psychiatry.

[25] Baeza-Velasco C., Cohen D., Hamonet C., Vlamynck E., Diaz L., Cravero C., Cappe E., Guinchat V. (2018). Autism, Joint Hypermobility-Related Disorders and Pain. Front. Psychiatry.

[26] Romeo D.M., Moro M., Pezone M., Venezia I., Mirra F., De Biase M., Polo A., Turrini I., Lala M.R., Velli C. (2023). Relationship and New Prospectives in Joint Hypermobility in Children with Autism Spectrum Disorder: Preliminary Data. J. Pers. Med..

[27] Manfredi F., Riefoli F., Coviello M., Dibello D. (2022). The Management of Toe Walking in Children with Autism Spectrum Disorder: “Cast and Go”. Children.

[28] Engelbert R., Gorter J.W., Uiterwaal C., van de Putte E., Helders P. (2011). Idiopathic toe-walking in children, adolescents and young adults: A matter of local or generalised stiffness?. BMC Musculoskelet. Disord..

[29] Sobhani-Eraghi A., Motalebi M., Sarreshtehdari S., Molazem-Sanandaji B., Hasanlu Z. (2020). Prevalence of joint hypermobility in children and adolescents: A systematic review and meta-analysis. J. Res. Med. Sci..

[30] Clinch J., Deere K., Sayers A., Palmer S., Riddoch C., Tobias J.H., Clark E.M. (2011). Epidemiology of generalised joint laxity (hypermobility) in fourteen-year-old children from the UK: A population-based evaluation. Arthritis Rheum..

[31] Shumnalieva R., Kotov G., Monov S. (2023). Obesity-Related Knee Osteoarthritis-Current Concepts. Life.

[32] Zoccante L., Ciceri M.L., Chamitava L., Di Gennaro G., Cazzoletti L., Zanolin M.E., Darra F., Colizzi M. (2021). Postural Control in Childhood: Investigating the Neurodevelopmental Gradient Hypothesis. Int. J. Environ. Res. Public Health.

[33] Colizzi M., Ciceri M.L., Di Gennaro G., Morari B., Inglese A., Gandolfi M., Smania N., Zoccante L. (2020). Investigating Gait, Movement, and Coordination in Children with Neurodevelopmental Disorders: Is There a Role for Motor Abnormalities in Atypical Neurodevelopment?. Brain Sci..

